# A Standardized Approach to Quantitative Analysis of Nicotine in e-Liquids Based on Peak Purity Criteria Using High-Performance Liquid Chromatography

**DOI:** 10.1155/2018/1720375

**Published:** 2018-08-09

**Authors:** Vinit V. Gholap, Leon Kosmider, Matthew S. Halquist

**Affiliations:** ^1^Department of Pharmaceutics, School of Pharmacy, Virginia Commonwealth University, Richmond, VA 23298, USA; ^2^Center for the Study of Tobacco Products, Virginia Commonwealth University, Richmond, VA 23298, USA

## Abstract

The use of electronic cigarettes (e-cigarettes) is a growing trend in population. E-cigarettes are evolving at a rapid rate with variety of battery powered devices and combustible nicotine refills such as e-liquids. In contrast to conventional cigarettes which are studied well for their toxicity and health effects, long-term clinical data on e-cigarettes are not available yet. Therefore, safety of e-cigarettes is still a major concern. Although the Food and Drug Administration (FDA) has recently started regulating e-cigarette products, no limits on nicotine and other ingredients in such products have been proposed. Considering the regulatory requirements, it is critical that reliable and standardized analytical methods for analyzing nicotine and other ingredients in e-cigarette products such as e-liquids are available. Here, we are reporting a fully validated high-performance liquid chromatography (HPLC) method based on nicotine peak purity for accurately quantifying nicotine in various e-liquids. The method has been validated as per ICH Q2(R1) and USP <1225> guidelines. The method is specific, precise, accurate, and linear to analyze nicotine in e-liquids with 1 to >50 mg/mL of nicotine. Additionally, the method has been proven robust and flexible for parameters such as change in flow rate, column oven temperature, and organic phase composition, which proves applicability of the method over wide variety of e-liquids in market.

## 1. Introduction

The growing popularity of e-cigarettes and vast number of available choices of e-liquids necessitate stringent analytical measurements and controls of these products for quality and regulations. As evolution of e-cigarettes is underway, a large number of e-liquids are being introduced in market [1]. A study carried out on the online market of e-cigarettes by Zhu et al. in [1] showed that there are more than 7700 e-liquid flavors available to customers. Such market is mainly Internet market where there is least control on quality and sale of e-cigarette products [2]. In past few years, several public health organizations and policy makers have expressed concerns over the safety and health impact of e-cigarettes [3, 4]. As a result, effective from August 8, 2016, all products meeting the statutory definition of tobacco products as per Tobacco Control Act are subject to regulations by Food and Drug Administration [5]. Such products also include e-cigarettes and e-liquids [5]. Although e-liquids are now subject to FDA regulations, guidelines and permissible limits of nicotine and other ingredients of e-liquids have not been finalized yet due to several reasons such as lack of definitive and long-term clinical data, assessment of safety claims about e-cigarettes [6–8], and standardized analytical methods [9]. The nicotine in e-liquid products is addictive and can be toxic in high doses [10]. Previous studies have shown that nicotine content in many e-liquids is highly variable than what is mentioned on label claim [11, 12].

Currently, there are several published methods to measure nicotine in e-liquids using gas chromatography with flame ionization detector (GC-FID), gas chromatography-mass spectrometry (GC-MS), and liquid chromatography-mass spectrometry (LC-MS) [13–16]. In contrast, there are few HPLC methods published for measuring nicotine in e-liquids. In Table 1, a summary of current available HPLC methods for measuring nicotine in e-liquids with associated validation parameters and shortcomings is assessed.

GC and mass analyzers are not available across all laboratories and may not be suitable for routine e-liquid analysis. HPLC methods published have several shortcomings as described in Table 1. To address these concerns, we report a standardized and alternative analytical HPLC method for quantification of nicotine in e-liquids of various flavors. As stated above, e-liquids contain a variety of ingredients other than nicotine. Therefore, it become necessary to accurately quantify nicotine without interference from other flavoring ingredients. As mentioned earlier, few HPLC methods have been published for quantification of nicotine in e-liquids. However, none of the method describes the peak purity of nicotine and also do not provide data for full validation of methods. Lack of reliable validation parameters questions the accuracy of data obtained and conclusions derived by such methods [9].

The current paper focuses on quantitative analysis of nicotine based on the peak purity criteria of nicotine using photo diode array (PDA) detector. Peak purity is an algorithm in the chromatographic software. It is analysis of absorbance spectra across a peak to determine similarity or differences between them. Differences in the spectra across a peak indicate that two more compounds are eluting at the same retention time. A peak is said to be pure if its purity angle is less than purity threshold [19]. Although peak purity may not serve as a full proof for chemical purity especially in active pharmaceutical ingredients (API), it serves as an important passing criterion for analytes in formulations. Besides, peak purity criterion is recommended by FDA and commonly used in pharmaceutical industries for analytes in formulations separated by chromatographic methods such as HPLC [19, 20].

The objective of this research was to develop and validate a HPLC analytical method for nicotine analysis in e-liquids. Additionally, taking into consideration the variety of flavoring ingredients in e-liquids, we also aimed to perform robustness study of the method with change in organic phase composition to achieve nicotine peak purity and optimum resolution (≥1.5) [21, 22] between nicotine and other ingredients.

## 2. Materials and Methods

### 2.1. Instrumentation

Method development and validation activities were carried out using a Waters Alliance 2695 quaternary pump HPLC equipped with Waters 996 PDA detector, Hypersil Gold Phenyl column (150 mm × 4.6 mm, 3 *µ*m, Thermo Scientific™, USA) and a Security Guard Cartridge Phenyl (4 mm × 2.0 mm, Phenomenex, USA). Waters Empower 2 software was used for processing data.

### 2.2. Chemicals and Reagents

Nicotine hydrogen tartrate standard (purity 93.14%) was purchased from Glentham Life Sciences, United Kingdom. Nicotine liquid standard (purity ≥99%) was purchased from Sigma-Aldrich, USA. HPLC grade acetonitrile, methanol, and water were purchased from BDH Chemicals, VWR, USA. Orthophosphoric acid (85%) was purchased from Merck, USA. Triethyl amine and hydrochloric acid (37%) were purchased from Sigma-Aldrich, USA. Sodium hydroxide (10 N) and hydrogen peroxide (30%) were purchased from BDH Chemicals, VWR, USA. Propylene glycol was purchased from Amresco LLC, VWR, USA. USP grade vegetable glycerin was purchased from JT Baker, USA. e-Liquids without nicotine (placebo) with variety of flavors of major categories were used. One e-liquid from each category of tobacco, vanilla, and two e-liquids from fruit flavors were purchased from Avail Vapor, USA. Eight other e-liquids were purchased from Direct Vapor online vape shop, USA, such as two e-liquids from each category of menthol, sweet, tobacco, and one from each category of fruit and coffee flavors. All e-liquids were coded with letters for the analysis.

### 2.3. Chromatographic Conditions

A gradient method was developed using an HPLC equipped with a quaternary pump system. Mobile phase A was 0.1% (v/v) triethyl amine in water with pH adjusted to 7.6 ± 0.05 by orthophosphoric acid (85%) and sodium hydroxide solution (1 N). Mobile phases B and C were 0.1% (v/v) triethyl amine in methanol and acetonitrile, respectively. Mobile phase D and diluent were 80% (v/v) methanol in water. The chromatographic conditions were run as shown in Tables 2 and 3. Peak purity analysis of nicotine peak was performed using threshold as “noise + solvent angle” calculated from blank and standard response using PDA detector (230 nm–350 nm).

### 2.4. Preparation of Reagents

Standard stock solution of nicotine hydrogen tartrate (1 mg/mL) in diluent was used for analysis. e-Liquids (8 mg/mL) were prepared by dissolving liquid nicotine standard in each flavored matrix. Similarly, quality control (QC) samples were prepared by dissolving liquid nicotine standard in unflavored matrix of propylene glycol and vegetable glycerin (1 : 1 v/v). Assay samples (80 *µ*g/mL) were prepared by 100-fold dilution of e-liquids and QC in diluent.

### 2.5. Method Validation

The method for nicotine quantification from e-liquids was validated as per ICH Q2(R1) and USP <1225> guidelines for specificity, linearity, accuracy, precision, LOD, LOQ, and robustness [20, 23].

#### 2.5.1. Specificity

Specificity of the method for nicotine quantification was established by performing forced degradation studies on various e-liquid assay samples, placebos, and blanks. Samples were subjected to acid hydrolysis, base hydrolysis, oxidation, and thermal degradation as mentioned in Table 4. Stressed samples were analyzed for % degradation, nicotine peak purity, and any degradant peak of nicotine.

#### 2.5.2. Linearity

Linearity of the method was established over the range of 0.4 *µ*g/mL to 500 *µ*g/mL of nicotine. Two sets of nicotine standard levels were prepared at 0.4, 10, 50, 100, 200, and 500 *µ*g/mL concentrations for generation of the calibration curve. The residual percent of nicotine was calculated by using the equation of the best fit line. Linearity was evaluated by linear equation, coefficient of variation (*r*^2^), and *Y*-intercept.

#### 2.5.3. Accuracy

Accuracy was established by analyzing standard and QC samples three times at 50%, 100%, and 150% of the assay level (80 *µ*g/mL). The averages of the results were calculated against the respective averages of standards prepared at approximately the same concentrations. Equation (1) was used to calculate % nicotine recovery:

(1) $$\begin{array}{c} \%\text{nicotine recovery}=\left(\frac{R\text{u}}{R\text{s}}\right)\times \left(\frac{C\text{s}}{C\text{u}}\right)\times 100, \end{array}$$

where *R*u = peak area of 50%, 100%, or 150% assay level, *R*s = average peak area of standard preparations of respective assay level, *C*s = concentration of standard preparation, and *C*u = concentration of assay level.

#### 2.5.4. Precision

Precision was expressed as the standard deviation or degree of reproducibility or repeatability of the analytical method under normal operating conditions. The % relative standard deviation (% RSD) of nicotine at 50%, 100%, and 150% accuracy samples was calculated to determine repeatability. Intermediate precision was performed by doing repeatability test by a different analyst on a different day. The % RSD of combined results obtained by both analysts was calculated to determine intermediate precision.

#### 2.5.5. Robustness

Robustness of the method performed by analyzing QC sample at assay level (*n*=3) by making minor changes to the method is as mentioned below:

- (±) 10% flow rate adjustment
- (±) 2°C column temperature adjustment
- Change in organic mobile phase ratio to modify polarity (Table 5).

Robustness was evaluated by calculating % RSD of replicate injections at each modified parameter.

#### 2.5.6. LOD and LOQ

Limit of detection (LOD) was calculated based on the standard deviation of the response and the slope. The detection limit (DL) is expressed as mentioned in the following equation:

(2) $$\begin{array}{c} \text{DL}=3.3\left(\frac{\sigma }{S}\right), \end{array}$$

where *σ* = standard deviation of the response and *S* = slope of calibration curve. *σ* was calculated as standard deviation of analytical background response at the retention time of nicotine and obtained from three placebo samples.

Limit of quantitation (LOQ) was calculated based on visual evaluation where minimum known concentration of nicotine can be analyzed quantitatively with acceptable accuracy and precision.

## 3. Results and Discussion

### 3.1. Method Development and Optimization

Based on published literature, earlier HPLC analytical methods have been developed for analysis of nicotine from e-liquids by reversed phase chromatography using a C18 column [11, 12, 18]. Some references for LC-MS method also describe use of a C18 column for separation of nicotine from other ingredients in e-liquids [13, 14]. Therefore, a C18 column was initially used for nicotine analysis. Mobile phase A was water with 0.1% TEA, pH adjusted to 7.6 ± 0.05, and mobile phase B was acetonitrile with 0.1% TEA in isocratic ratio of 70 : 30 (% v/v). Various e-liquids were analyzed for nicotine content. Although nicotine eluted as a single peak in a chromatogram, spectral scans of the nicotine peak were found to be impure in many of the e-liquids such as tobacco and fruit flavors. Several chromatographic runs were performed by varying parameters such as mobile phase composition, pH, and organic phase. However, feasibility of C18 column to separate nicotine peak from multiple flavors of e-liquids was found to be limited in terms of achieving peak purity for nicotine peak. Therefore, the objective of HPLC method development and optimization was based on achieving acceptable peak purity for nicotine from various e-liquid samples using a robust and flexible method.

Since e-liquids contain a variety of flavoring agents (>7700) [1] covering a wide range of chemicals such as unsaturated, aromatic, polycyclic, and so on; phenyl column chemistry was chosen for separation of these compounds from nicotine. Varying compositions of mobile phases, pH, column temperature, and flow rate were carried out using a phenyl column (250 mm × 4.6, 5 *µ*m).

Optimization of the chromatographic parameters was performed using Hypersil Gold Phenyl (150 mm × 4.6 mm, 3 *µ*m) to decrease the run time. Since e-liquids are complex mixtures of compounds, high organic solvent composition was used for elution of late eluting peaks. The proposed method has a run time of 12 min with the nicotine retention time at approximately 5.5 min with a postelution wash. Twelve different flavors of e-liquids from six major categories as mentioned in Section 2.2 were tested using our proposed method. Both methanol and acetonitrile (mobile phase B and mobile phase C) were used to achieve optimum polarity (and resolution) for separation of flavoring agents from nicotine. In all the twelve e-liquids, the nicotine peak was found to be pure.

Since flavoring agents are composed of a variety of chemicals, a fixed composition of mobile phase may not work for separation of nicotine from all flavoring agents. To address this hypothesis, we performed a study on the effects of change in organic phase composition on separation of flavoring agents from nicotine. Based on observations as described in Section 3.6, we propose that a variation in the ratio of mobile phase B (methanol) and C (acetonitrile) provides a window (±10%) for changing the organic polarity to achieve optimum resolution between interfering flavoring agents, if any, and nicotine. The run time can be extended, if required, for optimum postelution phase after each run. The proposed method has been validated for this flexibility and robustness as described in Section 3.6.

### 3.2. Specificity- and Stability-Indicating Study

Specificity in HPLC analysis is the ability to assess an analyte in the presence of other components in the sample matrix such as impurities, degradation product, and excipients. Forced degradation studies were carried out to test the specificity of the method for the nicotine peak. Ten e-liquid flavors were subjected to various stress conditions such as acid and base hydrolysis, oxidation, and thermal degradation. All samples were checked for peak purity of nicotine and any degradant peak of nicotine. Results of the forced degradation study are as mentioned in Table 6.

Nicotine content from each control sample of each flavor was found to be within the range of 90–110% of labeled claim. Samples were subjected to various stress conditions and compared against their respective control assays. The e-liquid flavors have different compositions; therefore, the respective % degradation was found to be different.

Acid and base hydrolysis with 0.1 N hydrochloric acid and 1 N sodium hydroxide, respectively, for 30 min was found to cause more than 5% degradation in one fruit flavored and one tobacco flavored e-liquid, respectively. The nicotine peak was found to pass the peak purity criteria in all samples with base to base separation of the nicotine peak.

Oxidation of e-liquid flavors was carried out with 6% peroxide for 30 min. The percent degradation was found to be more than 5% in each of sweet, tobacco, menthol, and fruit flavored e-liquids. In all oxidation degradations, nicotine peak was found to pass peak purity criteria with base to base separation of nicotine peak.

A standard nicotine solution was found to give more than 5% degradation in all three conditions.

Thermal degradation was carried out at 60°C for 2 hrs. The percent assay values obtained for the thermal degradation was found to be higher than control assays. Thermal degradation was found to concentrate samples possibly due to evaporation of solvent. Therefore, thermal degradation was not considered for specificity.

**Figure 1:**
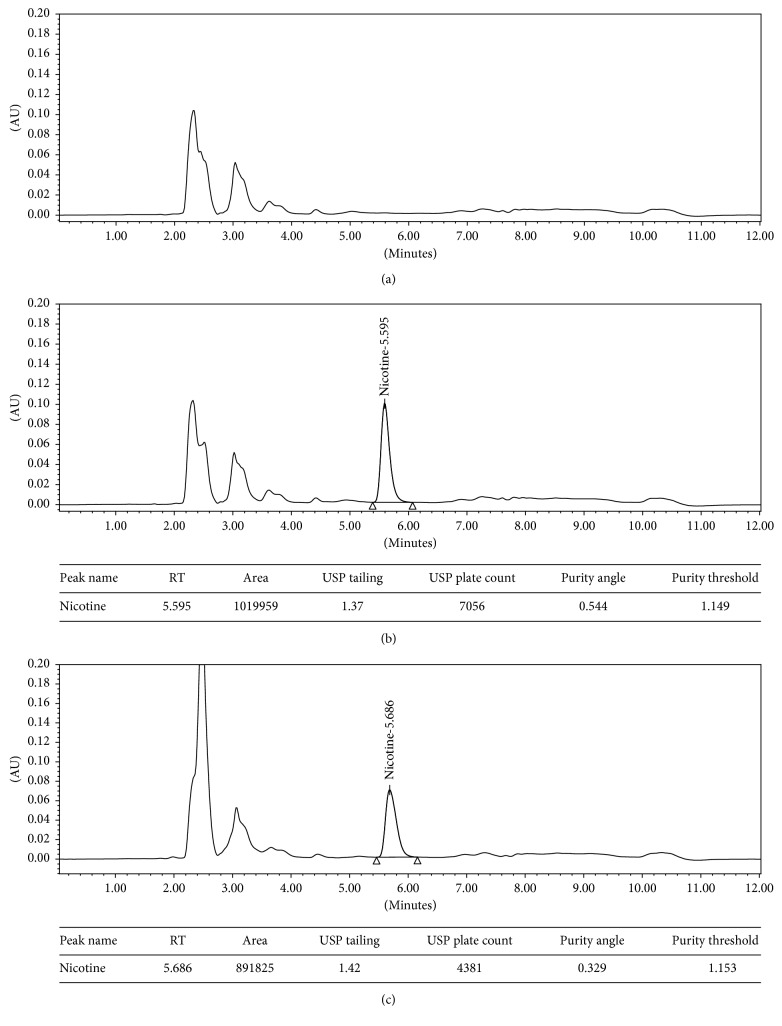
(a) Representative chromatogram of e-liquid flavor P, a placebo_control. (b) Representative chromatogram of e-liquid flavor P, a sample control. (c) Representative chromatogram of e-liquid flavor P, a sample_oxidation (peroxide degradation).

In conclusion, more than 5% degradation was observed in each of menthol, tobacco, fruit, and sweet flavored e-liquids. In all the degradation patterns, the nicotine peak was found to pass peak purity. No nicotine degradant peaks were observed at specified nicotine absorbance wavelength (260 nm). ICH and USP guidelines for validation and stability testing do not specify the limits of degradation in forced degradation studies. A degradation of 5–20% of analyte in at least one of the stressed conditions is generally the accepted range of forced degradation [24, 25]. Based on the degradation pattern observed, the HPLC method was found to be specific and stable indicating for nicotine. Representative chromatograms are shown in Figure 1.

### 3.3. Linearity and Range

Linearity is a measure of accuracy over the range of the method. e-Liquids are available in market with nicotine concentration ranging from 1 to >50 mg/mL. The proposed method is based on “dilute (100-fold) and inject” sample preparation approach. Therefore, linearity of the current method was established in the range of 0.4–500 *µ*g/mL which would cover the wide range of nicotine concentration in e-liquids available in market. Linearity was measured by calibration curve of the nicotine standard. The method was found to be linear over the specified range with linear equation *Y* = 13000 *X−*266. The coefficient of variation was found to be *R*^2^ = 1.000 with *Y*-intercept less than 3.0% of the peak area of the assay sample.

### 3.4. Accuracy

The accuracy of an analytical method is the closeness of test results obtained by that method to the theoretical or labeled value. Accuracy is expressed as percent recovery of known, added amounts of analyte. The results of accuracy parameter of the method are shown in Table 7. The % recovery of nicotine at 50%, 100%, and 150% of assay level was found to be within 98 to 102% with % RSD of triplicate preparations NMT 2.0.

### 3.5. Precision

The precision of an analytical method is the degree of agreement among individual test results when the procedure is applied repeatedly to multiple samplings of a homogenous sample. The precision of the method is expressed as repeatability under normal operating conditions.

The repeatability results were calculated from the accuracy samples as shown in Table 7. The % RSD of triplicate preparation at each level was found to be NMT 2.0.

Intermediate precision has been determined by repeatability of six preparations of assay samples across different days and different analysts under normal operating conditions. The combined % RSD of response of all twelve preparations was found to be NMT 2.0.

### 3.6. Robustness

Robustness of the method was evaluated as mentioned in Section 2.5.5. An assay sample was injected three times for each parameter of robustness. The % RSD of triplicate injections at each parameter was found to be no more than 2.0. Nicotine peak passed the peak purity criteria in all parameters.

**Figure 2:**
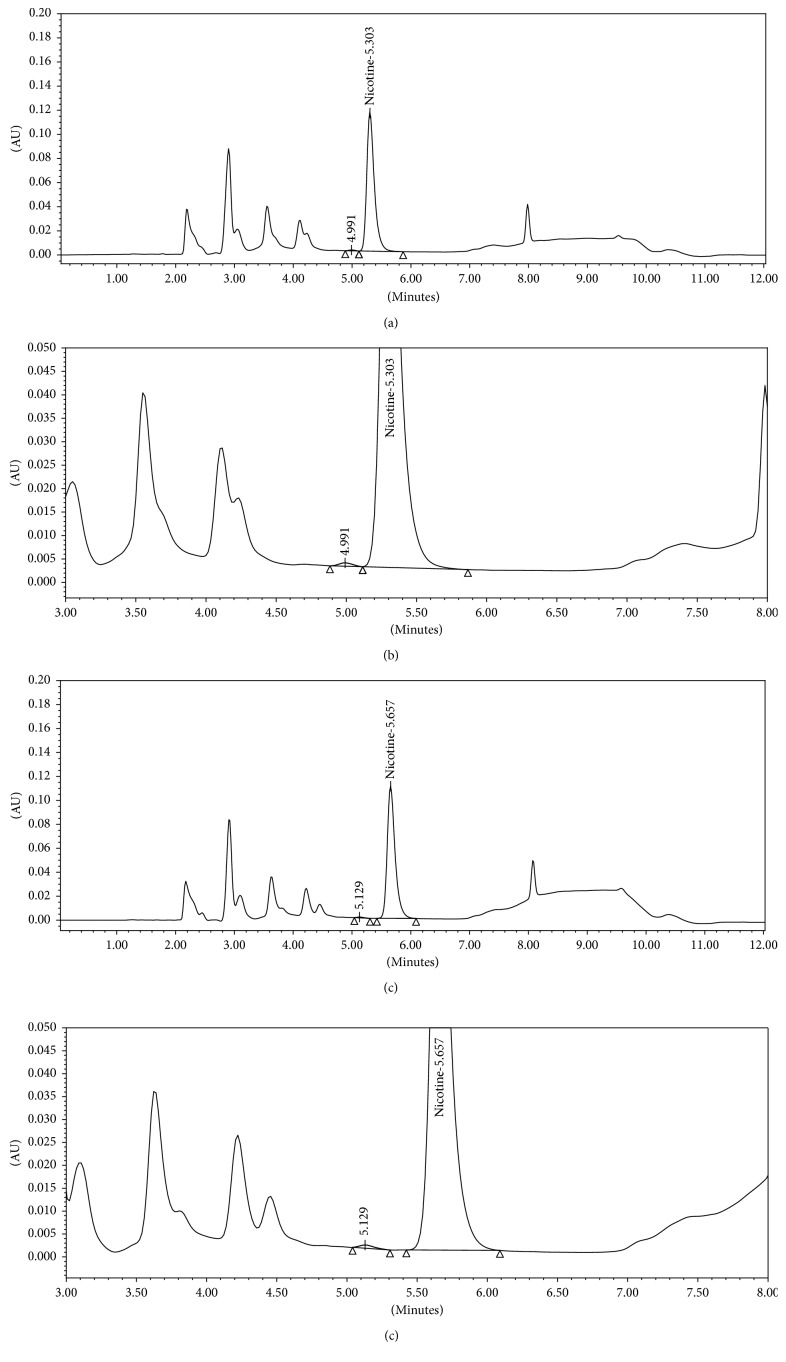
(a) Full scale representative chromatogram of e-liquid flavor V sample_mobile phase composition B : C, 26 : 14 v/v. (b) Zoomed representative chromatogram of e-liquid flavor V sample_mobile phase composition B : C, 26 : 14 v/v. (c) Full scale representative chromatogram of e-liquid flavor V sample_mobile phase composition B : C, 28 : 12 v/v. (d) Zoomed representative chromatogram of e-liquid flavor V sample_mobile phase composition B : C, 28 : 12 v/v.

**Figure 3:**
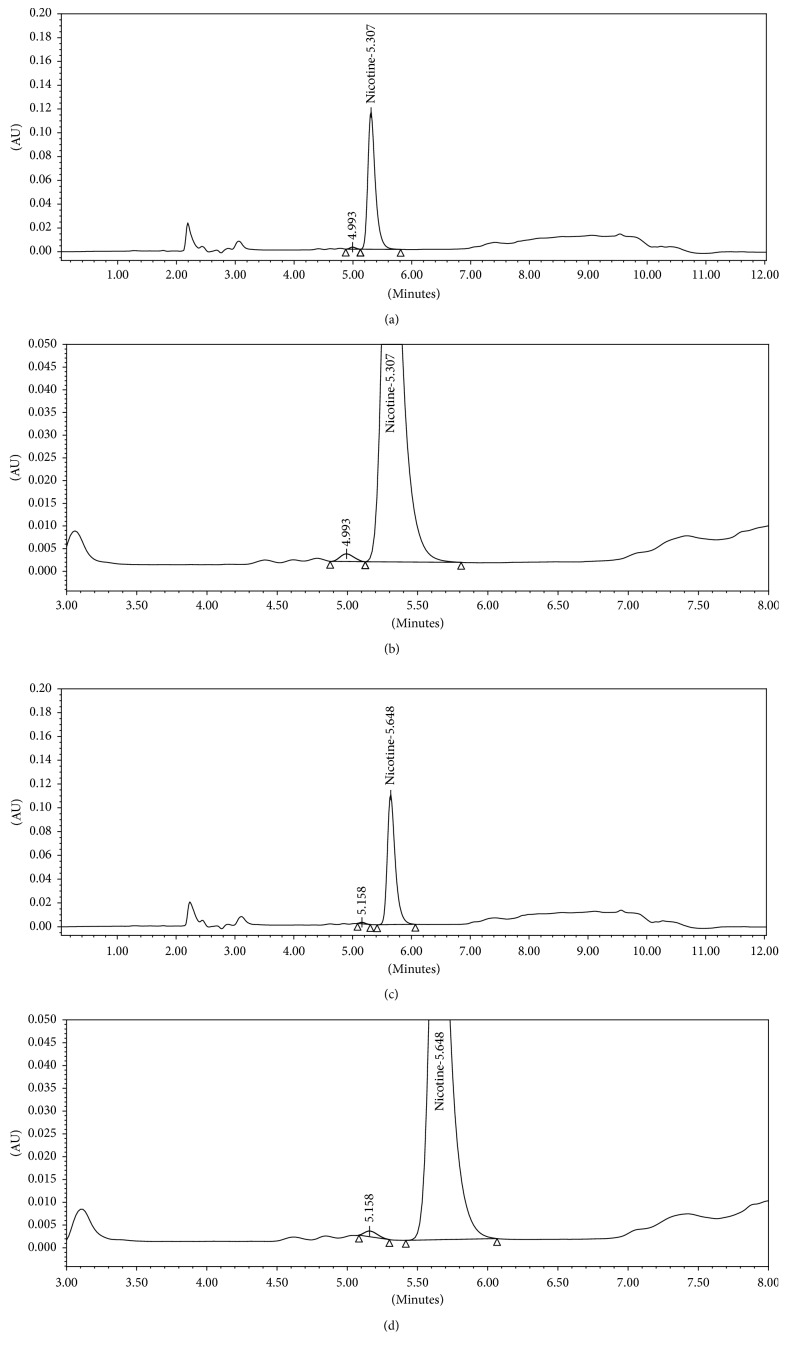
(a) Full scale representative chromatograph of e-liquid flavor W sample_mobile phase composition B : C, 26 : 14 v/v. (b) Zoomed representative chromatograph of e-liquid flavor W sample_mobile phase composition B : C, 26 : 14 v/v. (c) Full scale representative chromatograph of e-liquid flavor W sample_mobile phase composition B : C, 28 : 12 v/v. (d) Zoomed representative chromatograph of e-liquid flavor W sample_mobile phase composition B : C, 28 : 12 v/v.

In addition to the robustness evaluations, a separate study of the effects of change in organic phase composition on separation of flavoring agents from nicotine was carried out using two different e-liquid flavors V and W of two different categories tobacco and sweet, respectively, which were not tested for specificity study in the validation. The current method was found to be able to separate the nicotine peak from flavoring agents of these two e-liquids, however, with resolution <USP 1.5. ICH and USP recommendation for peak resolution is >2.0, and for accurate quantification, the resolution between peaks should be at least 1.5 [21, 22]. Therefore, to improve peak resolution, same samples were run using a robustness parameter of change in organic mobile phase ratio to modify organic polarity. With decrease in organic polarity, resolution between nicotine and adjacent peak was significantly improved. The results are mentioned in Table 8. Based on these results, it can be concluded that the proposed method can be optimized considering robustness parameter of change in organic phase composition to achieve optimum resolution and peak purity of nicotine peak in those e-liquid flavors which might show interference of flavoring agents at nicotine peak. Representative chromatograms are shown in Figures 2 and 3.

### 3.7. Limit of Detection and Limit of Quantification (LOD and LOQ)

Limit of Detection (LOD) was calculated based on the standard deviation of the response and the slope, as described in Section 2.5.6. The detection limit (DL) is expressed as shown in (2).

Based on the placebo response and linear equation of calibration curve (Section 3.3), LOD was found to be 0.07 *µ*g/mL of nicotine.

Limit of quantitation (LOQ) was calculated based on visual evaluation. The minimum known concentration of nicotine which can be analyzed quantitatively with acceptable accuracy and precision using current method was found to be 0.45 *µ*g/mL of nicotine. The accuracy and precision were performed at LOQ level (*n*=6). % recovery was found to be within 90 to 110% and % RSD of all six preparations was NMT 10.0. Results are mentioned in Table 9.

### 3.8. System Suitability

System suitability test was performed by running six injections of standard at assay level. % RSD of the response of six injections was found to be NMT 1.0. USP limits for theoretical plate count (NLT 2000) and peak tailing (NMT 2.0) were applied. The system suitability test of the current method passed all USP criteria (Table 10).

## 4. Conclusion

An alternative standardized HPLC method for analysis of nicotine in e-liquids has been developed. The method has been fully validated as per ICH and USP guidelines. The method is found to be specific for nicotine as determined by peak purity criteria. The method has been tested for stability, accuracy, and precision for quantification of nicotine in e-liquids. Linearity of the method has been achieved over a wide range of 0.4 to 500 *µ*g/ml of nicotine concentration. Thus, the method can be used to measure e-liquids with concentration ranging from 1 to >50 mg/mL. Since e-liquids are available in the market with a variety of flavoring combinations, a fixed composition of mobile phase may not work for separation of nicotine from all flavoring agents. Therefore, we tested the method for robustness parameters of change in flow rate, column oven temperature, and organic phase composition. After achieving the nicotine peak purity by successfully passing the robustness parameters, we are concluding that the proposed HPLC method for analysis of nicotine in e-liquids is flexible with accuracy over a wide variety of e-liquids in the market.

## Figures and Tables

**Table 1:** HPLC methods published for analysis of nicotine in e-liquids.

Type of detection platform used	Validation parameters evaluated	e-Liquids (brand and number of samples)	Reference	Comments
HPLC, PDA, C 18 (150 × 4.6 mm, 5 *µ*m)	LOD, LOQ, linearity, accuracy, and precision	Smoking everywhere (15), Njoy (5), and CIXI (10)	Trehy et al. [12]	1. Specificity data are not mentioned
				2. Although the method uses PDA detector, no information about peak purity is mentioned
				3. Chromatographic integration in the chromatograms published by Trehy et al. is improper, which raises concerns over the specificity of the method
HPLC, PDA, C 18 (200 × 4.6 mm, 5 *µ*m)	LOD, LOQ, linearity, accuracy, and precision	Refill fluids (75), do it yourself (1)	Davis et al. And cross-reference of Trehy et al. For the HPLC method [11]	

UPLC	Not applicable	e-Liquids (20). Details not mentioned	Etter et al. [17]	Method not validated for analysis of e-liquids

UPLC, PDA and MS, C 18	Linearity	e-Liquids (6). Details not mentioned	Meruva et al. And cross-reference of Trehy et al. for the HPLC method [18]	1. Additional validation details not provided
				2. No data about specificity of the method for nicotine in presence of flavoring chemicals

**Table 2:** HPLC chromatographic conditions of the method.

Chromatographic conditions	
Flow rate	0.8 mL/min
Wavelength	260 nm
Stationary phase	Hypersil Gold Phenyl (150 mm × 4.6 mm, 3 *µ*m)
Column oven temperature	25°C
Injection volume	10 *µ*L
Sample cooler temperature	5°C
Run time	12 min

**Table 3:** HPLC gradient program.

Pump program				
Time (min)	A (%)	B (%)	C (%)	D (%)
0	60	26	14	0
4	60	26	14	0
4.1	0	0	0	100
7	0	0	0	100
7.1	60	26	14	0
12	60	26	14	0

**Table 4:** Stressed conditions for e-liquid assay samples, placebos, and blanks.

Sample stress type	Time	Assay sample (mL)	Water (mL)	0.1 N·HCl (mL)	1 N·NaOH (mL)	H_2_O_2_ (6%) (mL)
Control	N/A	4.5	0.5	0	0	0
Acid hydrolysis	30 min	4.5	0	0.5	0	0
Base hydrolysis	30 min	4.5	0	0	0.5	0
Oxidation	30 min	4.5	0	0	0	0.5
Thermal	2 hrs	4.5	0.5	0	0	0

**Table 5:** Change in organic mobile phase ratio.

Change parameter	% mobile phase A	% mobile phase B	% mobile phase C
Increase in organic polarity	60	24	16
Decrease in organic polarity	60	28	12

**Table 6:** Results of forced degradation of various e-liquid flavors.

Name of sample	Category	Stressed condition			
		% control assay	% acid degradation	% base degradation	% oxidation degradation
Standard	NA	NA	7.75	8.29	7.62
QC	NA	99.39	ND	ND	3.5
e-Liquid flavor P	Sweet	95.98	ND	ND	12.2
e-Liquid flavor Q	Menthol	100.76	1.53	ND	4.14
e-Liquid flavor R	Tobacco	103.09	4.69	5.68	7.53
e-Liquid flavor S	Menthol	100.14	2.04	3.38	10.28
e-Liquid flavor T	Fruit	96.18	1.03	ND	2.06
e-Liquid flavor U	Coffee	97.68	ND	1.59	4.33
e-Liquid flavor A	Tobacco	96.19	1.94	1.00	4.75
e-Liquid flavor C	Vanilla	97.31	1.38	0.69	4.44
e-Liquid flavor E	Fruit	98.13	ND	1.44	14.13
e-Liquid flavor G	Fruit	96.94	6.38	1.00	2.5

*Note.* % degradation of ±1% is considered as no degradation (ND).

**Table 7:** Accuracy results of the method.

% spiked level of assay sample	Replicate	% recovery	% mean recovery	% RSD
50 (40 *µ*g/mL)	1	99.95	99.44 (39.78 *µ*g/mL)	0.77
	2	99.81		
	3	98.56		

100 (80 *µ*g/mL)	1	99.41	100.01 (80.01 *µ*g/mL)	0.95
	2	99.52		
	3	101.11		

150 (120 *µ*g/mL)	1	100.27	100.61 (120.73 *µ*g/mL)	0.39
	2	101.04		
	3	100.51		

**Table 8:** Robustness.

Organic phase composition		Sample	Peak area	USP resolution	Purity angle	Purity threshold
% mobile phase B	% mobile phase C					
26	14	e-Liquid flavor V	1031106	1.46	0.729	1.159
			1031686	1.51	0.390	1.106
		e-Liquid flavor W	1023000	1.48	0.984	1.138
			1021702	1.48	0.915	1.144

28	12	e-Liquid flavor V	1030040	2.31	0.192	1.063
			1026349	2.36	0.471	1.104
		e-Liquid flavor W	1023658	2.30	0.134	1.093
			1027008	2.28	0.295	1.128

**Table 9:** Accuracy and precision at LOQ level.

% spiked level of assay sample	Replicate	% recovery	% mean recovery	% RSD
LOQ	1	90.89	97.08	6.78
	2	90.63		
	3	99.22		
	4	103.92		
	5	105.17		
	6	92.64		

**Table 10:** System suitability test.

Replicate injection	Area	USP tailing	USP plate count
A1	1299677	1.41	8979
A2	1295191	1.40	9211
A3	1297765	1.40	9132
A4	1294551	1.40	9225
A5	1291896	1.40	9194
A6	1289339	1.39	9035
Mean	1294737	1.40	9129
Std. dev.	3768.89	0.01	101.50
%RSD	0.29	0.45	1.11

*Note.* No adjacent peaks were observed at the retention time of nicotine. Hence USP resolution criteria is not applicable.

## Data Availability

The data used to support the findings of this study are available from the corresponding author upon request.

## Abbreviations

NMT: Not more than
NLT: Not less than
ND: Not detected
RSD: Relative standard deviation
LOD: Limit of detection
LOQ: Limit of quantitation.

## References

[1] Zhu S. H., Sun J. Y, Bonnevie E. (2014). Four hundred and sixty brands of e-cigarettes and counting: implications for product regulation. *Tobacco Control*.

[2] Noel J. K., Rees V. W., Connolly G. N. (2011). Electronic cigarettes: a new “tobacco” industry?. *Tobacco Control*.

[3] Bhatnagar A., Whitsel L. P., Ribisl K. M. (2014). Electronic cigarettes: a policy statement from the American Heart Association. *Circulation*.

[4] Blanding M. (2016). *The E-Cig Quandary|Harvard Public Health Magazine|Harvard T.H. Chan School of Public Health*.

[5] Food And Drug Administration (2017). *Guidance for Industry Listing of Ingredients in Tobacco Products*.

[6] Callahan-Lyon P. (2014). Electronic cigarettes: human health effects. *Tobacco Control*.

[7] Manzoli L., La Vecchia C., Flacco M. E. (2013). Multicentric cohort study on the long-term efficacy and safety of electronic cigarettes: study design and methodology. *BMC Public Health*.

[8] Drummond M. B., Upson D. (2014). Electronic cigarettes: potential harms and benefits. *Annals of the American Thoracic Society*.

[9] Kaisar M. A., Prasad S., Liles T., Cucullo L. (2016). A decade of e-cigarettes: limited research & unresolved safety concerns. *Toxicology*.

[10] Department of Health and Human Services U.S. (2010). *How Tobacco Smoke Causes Disease: The Biology and Behavioral Basis for Smoking-Attributable Disease*.

[11] Davis B., Dang M., Kim J., Talbot P. (2015). Nicotine concentrations in electronic cigarette refill and do-it-yourself fluids. *Nicotine & Tobacco Research*.

[12] Trehy M. L., Ye W., Hadwiger M. E. (2011). Analysis of electronic cigarette cartridges, refill solutions, and smoke for nicotine and nicotine related impurities. *Journal of Liquid Chromatography & Related Technologies*.

[13] Flora J. W., Wilkinson C. T., Sink K. M., McKinney D. L., Miller J. H. (2016). Nicotine-related impurities in e-cigarette cartridges and refill e-liquids. *Journal of Liquid Chromatography & Related Technologies*.

[14] Liu X., Joza P., Rickert B. (2017). Analysis of nicotine and nicotine-related compounds in electronic cigarette liquids and aerosols by liquid chromatography-tandem mass spectrometry. *Beiträge zur Tabakforschung International/Contributions to Tobacco Research*.

[15] Aszyk J., Kubica P., Kot-Wasik A., Namieśnik J., Wasik A. (2017). Comprehensive determination of flavouring additives and nicotine in e-cigarette refill solutions. Part I: liquid chromatography-tandem mass spectrometry analysis. *Journal of Chromatography A*.

[16] Herrington B. J. S., Myers C., Rigdon A. (2015). *Analysis of Nicotine and Impurities in Electronic Cigarette Solutions and Vapor*.

[17] Etter J. F., Zäther E., Svensson S. (2013). Analysis of refill liquids for electronic cigarettes. *Addiction*.

[18] Meruva N. K., Benvenuti M. E., Cleland G. E., Burgess J. A. (2016). *Simultaneous Determination of Nicotine and Related Impurities in E-Liquids and E-Cigarettes Using UPLC-UV-MS*.

[19] Waters Corporation (1998). *Waters 996 Photodiode Detector: Peak Purity I What is Peak Purity Analysis? Peak Purity Analysis*.

[20] International Conference on Harmonization (2005). *ICH Topic Q2 (R1) Validation of Analytical Procedures: Text and Methodology*.

[21] Bose A. (2014). HPLC calibration process parameters in terms of system suitability test. *Austin Chromatography*.

[22] The United States Pharmacopeia (2015). General chapter, <621> chromatography. *USP40-NF35*.

[23] The United States Pharmacopeia (2011). 1225 validation of compendial procedures. *USP34-NF29*.

[24] Alsante K. M., Ando A., Brown R. (2007). The role of degradant profiling in active pharmaceutical ingredients and drug products. *Advanced Drug Delivery Reviews*.

[25] Sharma M. K., Murugesan M. (2017). Forced degradation study an essential approach to develop stability indicating method. *Journal of Chromatography and Separation Techniques*.

